# The Impact of Service Dogs on Military Veterans and (Ex) First Aid Responders With Post-traumatic Stress Disorder

**DOI:** 10.3389/fpsyt.2022.834291

**Published:** 2022-05-04

**Authors:** Emmy A. E. van Houtert, T. Bas Rodenburg, Eric Vermetten, Nienke Endenburg

**Affiliations:** ^1^Animals in Science and Society, Faculty of Veterinary Medicine, Utrecht University, Utrecht, Netherlands; ^2^Department of Psychiatry, Leiden University Medical Centre, Leiden, Netherlands; ^3^Arq Psychotrauma Expert Group, Diemen, Netherlands; ^4^Department of Militaire Geestelijke Gezondheidszorg (MGGZ), Ministry of Defence, Utrecht, Netherlands

**Keywords:** service dog, PTSD, veteran, dog, post-traumatic stress disorder

## Abstract

Due to its novelty and lack of empirical study it remains unclear if a service dog truly mitigates the burden of post-traumatic stress disorder (PTSD) symptoms. To cross sectionally investigate the effect of service dogs on veterans and first aid responders with PTSD, we studied subjective and physiological parameters in 65 individuals divided over four groups. These groups were: veterans and first aid responders with PTSD and a service dog (*n* = 20), with PTSD and a companion dog (*n* = 10), with PTSD without a dog (*n* = 12) and a group without PTSD (*n* = 23). We found that veterans and first aid responders with PTSD who had a service dog showed significantly less PTSD related symptoms, better sleep quality, and better wellbeing experience, than those with a companion dog. Those with a service dog additionally experienced fewer PTSD related symptoms than those without a service dog and tended to walk more than individuals without PTSD. No differences were found in cortisol levels between groups though and changes in both salivary cortisol and activity were not linked to improved welfare experience. Though the use of physiological measurement methods thus warrants more research, our study indicates that the subjective experience of wellbeing, sleep quality and PTSD related symptoms is improved by the presence of a service dog.

## Introduction

PTSD in veterans typically is a chronic disorder, that manifests in a general negative mood, periods of depression, periods of anxiety, flashes of anger, reckless behavior, sleeplessness, and general increased arousal causing impairment or distress (1). A novel way in which the symptoms of PTSD can be lessened is through the provision of a PTSD service dog. Service dogs are specialized assistance animals, which have learned to respond to various verbal and non-verbal communication ques of their handler. As a response they will act as both a social support and behavioral mirror, and help their handler in coping with the consequences of their PTSD. Service dogs are further known to work as a social facilitator *via* their learned behaviors (2) and their presence as a companion animal (3–5). This function is related to the principles of Behavioral Activation (BA), which has been shown to be an effective treatment for depression (6, 7). Because there is also empirical support for BA as a treatment for PTSD (7), the principles of BA may provide evidence for PTSD service dog effect. The most compelling evidence of the effectiveness of service dogs to date however, seems to be in the form of self-report by those who are supported by a service dog (8–11). In these reports handlers state that their service dog helps them reclaim control of their life and obtain a sense of worth by promoting responsibility and self-efficacy through the care the service dog needs (12). Service dogs are further stated to help handlers reconnect with society, improve individual quality of life, and therefore help their handlers reach opportunities in life they previously deemed unreachable (2).

All these effects seem to speak in high favor of the provision of service dogs to individuals with PTSD. In our literature study from 2018 we however concluded that the presented evidence of service dog effectiveness at that time was insufficient to definitively attribute any improved wellbeing in individuals with PTSD to service dog presence (13). This attribution was difficult because, as stated above, the influence of service dogs is mostly measured *via* self-report measurements. Although very valuable in the determination of individual wellbeing, these measurements do not indicate physiological changes that might be influenced by both PTSD and presence of a PTSD service dog. We further determined that many studies on service dogs were conducted among small sample sizes, did not have control groups, and had vastly varying measurement methodologies, which made them difficult to compare to one another (13). All this led to the conclusion that further study regarding the effect of service dogs on individuals with PTSD needed to address the above uncertainties by not only introducing standardization in methodology, but also by introducing the use of quantifiable measurements to complement and frame the subjective experience of service dogs by individuals with PTSD.

One study which has since addressed some of the uncertainties in service dog research is that of Rodriguez et al. (14). In their study they compared the morning awakening cortisol response in 45 veterans with a service dog with that of 28 individuals on a waiting list to receive one. By doing so, they found that individuals with a service dog had a higher morning awakening cortisol level than those on the waiting list. Morning awakening cortisol is a measure related to the human circadian cortisol rhythm. In this rhythm a basal release of the hormone is regulated throughout the day by the suprachiasmatic nucleus in the hypothalamus (15, 16). In individuals with PTSD this basal release of cortisol is known to deviate from that of non-PTSD individuals. Though differences in the overall circadian average are disputable between groups (17), evening peak and early morning levels of cortisol were found to be lower in individuals with PTSD (18–20). The results of Rodriguez et al. (14) therefore suggest that the difference in presence or absence of a service dog between their two subject groups influenced the manner in which PTSD affected the subjects’ cortisol response, and brought the service dog group closer to what could be expected of non-PTSD afflicted individuals. If this conclusion is correct, they have provided one of the first measurements that can be used to quantify the influence of service dog presence on an individual with PTSD and have therefore created interest in the use of other PTSD symptom related measurement techniques in service dog research.

One of these other measurement techniques is through the observation of changes in behavioral patterns and overall functioning of individuals with PTSD (21). Although PTSD can express differently between individuals, it generally alters observable behavior and functioning in an individual compared to non-PTSD individuals. Especially overall activity and activity intensity are known to decrease in those with PTSD since they are less inclined to leave their house or safe environment. The degree in which an individual undertakes activities and is active in his or her daily life is could therefore be seen as an indicator of how he or she is affected by PTSD. Combined with a record of service dog presence, an individual’s activity level or changes therein can thus be used to evaluate the effect of the service dog on PTSD related symptoms (21).

All in all, there are various measurements with which the effect of PTSD on human physiology and psychology can be quantified. The objective of this study was therefore to identify the influence of a service dog on activity levels and morning salivary cortisol levels of individuals with PTSD. More specifically we asked several questions regarding these measurements. The first question was whether the presence or absence of a service dog is measurable in the 24 h activity pattern of individuals with PTSD? We hypothesized that individuals with PTSD and a service dog would be more active than those with PTSD without a dog, though not necessarily more than those with PTSD and a pet dog. Our second question concerned cortisol levels and whether or not the presence or absence of a service dog is measurable in the morning and evening cortisol of individuals with PTSD. Our hypothesis for this question was that individuals with PTSD and a service dog would approach cortisol levels expressed by individuals without PTSD, while levels in those with PTSD without a service dog would deviate. Our third question finally was whether or not the morning waking cortisol and 24 h activity pattern of those with PTSD were positively correlated to wellbeing experience as reported. Our hypothesis for this question was that individuals with PTSD who evaluate their own wellbeing the highest also are the most active and have cortisol levels that approach those of individuals without PTSD, If these questions could be answered, they could provide insight in the effects that provision of a service dog might have on individuals with PTSD.

## Materials and Methods

### Subjects

Four groups were identified for this study (Total *n* = 65). The first group (*n* = 20) consisted of military veterans or (ex) first aid responders (ambulance workers, firefighters, police officers) who were currently matched with a service dog from the service dog provider Stichting Hulphond Nederland. We chose to only work with individuals who had received a service dogs from a single provider as to eliminate the influence of different training, education, selection, and support strategies on the performance of service dogs as an extra variable in this study. It was further chosen to only work with veterans or (ex) first aid responders with PTSD, as the origin, development, and support offered for PTSD is relatively similar between individuals in this group.

The second group (*n* = 12) consisted of military veterans or (ex) first aid responders with PTSD who were currently waiting to be matched with a service dog from the abovementioned service dog provider. Individuals in the second group were additionally not in the possession of a companion dog, as those who already had a companion dog (besides waiting for a trained service dog) were considered a separate third group (*n* = 10). This division between groups two and three was made to see if the presence of a companion dog had a positive influence on veterans/(ex) first aid responders with PTSD, and if so, to see if this influence was different from the influence of a service dog. The fourth and final group of participants (*n* = 23) consisted of military veterans without PTSD. Details of each group can be found in Table 1.

**TABLE 1:** Details on the participants of the four subject groups in this study.

Group	Male	Female	Age	Veteran*	First aid responder*	PTSD	Service dog
							
	%	%	Years	%	%	%	%
1	90	10	52	84	47	100	100
2	75	25	20	80	58	100	0
3	100	0	47	67	60	100	0
4	91	9	51	100	26	0	0

*The first group consisted of veterans/(ex) first-aid responders with PTSD and a service dog (n = 20), the second group consisted of veterans/(ex) first-aid responders with PTSD but without any dog (companion or service dog) (n = 12), the third group consisted of veterans/(ex) first-aid responders with PTSD and a companion dog (n = 10), and the fourth group consisted of veterans without PTSD (n = 23).*

*The percentages of veterans and first aid responders do not add up because some participants were part of both groups (e.g., veterans who joined the police force after their deployment).

Contact with potential participants to the study was sought *via* various channels. All individuals of group one were contacted *via* the above mentioned service dog provider. This was also done for a number of individuals belonging to groups two and three who were on a waiting list to receive a service dog from that same service dog provider. The remaining participants in groups two, three, and four were finally found *via* a mixture of personal connections, and communication channels targeted at veterans.

### Experimental Design

All participants were instructed to perform several measurements at home. These measurements were: collecting 10 salivary samples at set timepoints over the course of 2 days, wearing an accelerometer for a period of 36 h, and filling out a maximum of five questionnaires. Individuals who had a service dog finally also collected 10 salivary swabs from their dog, made sure it wore an activity measuring collar and filled out an additional questionnaire. These dog based measurements were used for a study on service dog welfare. The results and full design of this study will be published separately.

To ensure the instructions for home measurements were clear, a researcher visited each participant in their home and explained every measurement before handing over the necessary equipment to perform them. This same researcher collected the used equipment after a period of at least a week, and answered any questions the participants might ask before, during, and after their participation to the study.

### Questionnaires

The five questionnaires used during this study were filled out by all subjects in all groups as long as the questionnaire was applicable to their situation. This means that questionnaires regarding dogs were not filled out by subjects without a dog. The five questionnaires were:

• An intake questionnaire used to register general information on each subject like age, sex, whether they were a veteran or (ex) first aid responder, whether or not they were diagnosed with PTSD, and if so whether they were assisted by a service dog or not.
• The PTSD Check List—version DSM 5 (PCL-5). The PCL-5 is a 20 item questionnaire concerning the prevalence or severity of trauma associated symptoms in individuals. Each answer can be given on a 5-point scale which indicates increasing prevalence or severity. If points for all answers are combined a score between zero and 80 points should be achieved (22), with a cut-off point at 31–33 points for PTSD diagnosis (23). Analysis of this questionnaire was performed *via* its included instructions which resulted in four component scores and a final score for each questionnaire.
• The Pittsburg Sleep Quality Index (PSQI) questionnaire. The PSQI is a 21 item self-report questionnaire which questions the frequency of disruptive nocturnal behaviors (DNB). It is made up of seven components; subjective sleep quality, sleep latency, habitual sleep efficiency, sleep duration, sleep disturbance, use of sleep medication and daytime functioning (24, 25). Analysis of this questionnaire was performed *via* its included instructions which resulted in a final score.
• The 36-Item Short Form Survey Instrument (SF36) questionnaire. The SF36 is a 36-item self-reflective wellbeing measurement tool with multiple choice answer format. Shiner et al. (26) found the SF36 to reproduce reliable results when filled out by a subject group of military veterans with PTSD. It was later also applied by Stern and Chur-Hansen (8) to evaluate experienced quality of life by military veterans in relation to service dog intervention. Analysis of this questionnaire was performed by first mirroring negative question scores before adding all answer scores into a final score.
• A Dutch translation of the Monasch Dog Owner Relationship Score (MDORS) (27, 28). The Dutch translation of the MDORS has 16 items divided over 2 factors; perceived emotional closeness and perceived costs of dog ownership. Answers can be given on a five-point multiple choice format which produces a score between 16 and 80 points. Analysis of this questionnaire was performed by first mirroring the score of negative questions before adding all answer scores into a final score. The MDORS questionnaire was only filled out by the participants that either had a service dog or a pet dog.

### Salivary Cortisol

To study deviations in normal morning and evening peripheral cortisol level between subject groups, salivary cortisol level was measured on 10 occasions divided over 2 days. On the first day, the first sample was taken in the morning directly after waking up. The next sample was taken 15 min later, the third 30 min after waking up, and the fourth 60 min after waking up. The fifth and final sample was taken right before going to sleep, after which the whole procedure was repeated the next morning and evening.

Sample collection by participants occurred in their individual home environment through passive drooling into a new collection tube at each time point. Participants were instructed not to eat, smoke, or consume any other fluid than clear water 30 min before each measurement, as this might influence sample quality (19). After collection, a sample was marked with its order-number and stored at −20°C until retrieval by the researcher. Retrieved samples were then transported to the general storage facility at Utrecht University, where they were again stored at −20°C.

Extraction of cortisol from samples was performed by spinning the samples at 3,000 rpm for 5 min. This resulted in a clear supernatant of low viscosity. Visual inspection was performed at this stage for any signs of contamination (discoloration). No samples were rejected because of this. Cortisol concentrations were finally measured using a commercially available chemiluminescence immunoassay with high sensitivity (IBL International, Hamburg, Germany). The average intra-assay coefficient was 5%.

The 10 samples over 2 days were finally reduced to five datapoints. This was done by averaging the sample of day 1 and day 2 for each time point. This resulted in a total of five datapoints per participant, one for each timepoint. If a participant missed a measurement on either 1 of the 2 days the final datapoint was based on a single measurement instead.

### Activity Measurements

Overall activity in all human subjects was monitored *via* the Empatica E4. Participants wore the E4 for a continuous period of at least 36 h. During those 36 h the device had to be worn at all time, both while awake and while sleeping, unless there was a high chance of damage to the devices (showering, swimming, working heavy tools). All registered data were stored on the device’s internal storage capacity until extraction *via* Empatica’s specialized E4 software. Data analysis was performed *via* the EDA explorer scripts of Taylor et al. (29). This entailed that each dataset was run through a step detection script which returned the estimated total number of steps, mean step time during movement and percentage of time spent inactive during the first 24 h.

### Statistical Analysis

Statistical analysis was performed in R version 4.0.3 with R studios (30). A total of 12 numeric variables were analyzed for differences between four participant groups. The variables were: salivary cortisol levels at five different timepoints, the number of steps taken in a 24 h time period, the mean time spent walking in a 24 h time period, the percentage of time an individual was inactive in a 24 h time period, an individual’s PCL5 questionnaire score, an individual’s PSQI questionnaire score, an individual’s SF36 questionnaire score, and an individual’s MDORS questionnaire score. For all these variables normality was judged by plotting a histogram and observing if the resulting figure neared normal distribution. From these histograms it became apparent that normality could not be assumed for any of the variables. A choice was therefore made for statistical analysis *via* non-parametric methods. This analysis was started with a Levene’s test of homogeneity of variances for each variable. None of these tests were significant which meant that equal variances could be assumed. To check if pairs of two participant groups differed from one another, a series of Mann-Whitney tests was performed per variable (α = 0.05). This resulted in six Mann-Whitney tests per variable according to the following schedule: group 1–2, 1–3, 1–4, 2–3, 2–4, 3–4. Significant results of these tests are represented in the results section *via* a *p*-value and an effect size r. r is based on the z scores of the Mann-Whitney tests (formula *r* = z/√N). Effect sizes around 0.8 are considered large, around 0.5 medium, and around 0.2 small (31).

A series of Spearman correlations (α = 0.05) was finally used to evaluate possible correlations between the questionnaire scores (PCL5, PSQI, SF36, MDORS) and the other variables.

### Ethics Statement

Ethical review and approval for this study was obtained from the medical ethical committee of the Utrecht Medical Centre, Utrecht, The Netherlands under number NL64117.041.18. Each participant further gave informed consent before participation to the study.

## Results

### Dataset Description

All but a few datasets were fully complete. This resulted in a reduced n for several measurements compared to the total participant number. Regarding salivary cortisol, a total of 60 out of 65 participants had at least one sample at all five timepoints. A further three had a sample for at least four timepoints, one had a sample for three timepoints, and one missed samples for all timepoints. The main reason for these missing samples was insufficient saliva volume.

Activity measurements were successful for 47 out of 65 participants. The most common reason for activity measurement to fail was due to (premature) battery failure of the measurement equipment.

The full set of questionnaires was finally retrieved for 55 out of 65 participants. The intake questionnaire missed one or more items for three participants, the PCL5 questionnaire missed one or more items for one participant, the PSQI missed one or more items for nine participants, and the SF36 questionnaire missed one or more items for two participants. Regarding the PSQI, missing values were often caused by one missing answer out of the total 21 items on the questionnaire. This missing item prevented a total score from being calculated, resulting in a missing value. To reduce the number of missing values for this questionnaire we decided to substitute the missing item, in questionnaires with only a single missing item, with the average of the treatment group that particular participant was assigned to. This substitution made it possible to calculate a PSQI score for an additional seven participants leaving only two fully missing datapoints.

### Salivary Cortisol Differences Between Groups

**FIGURE 1:**
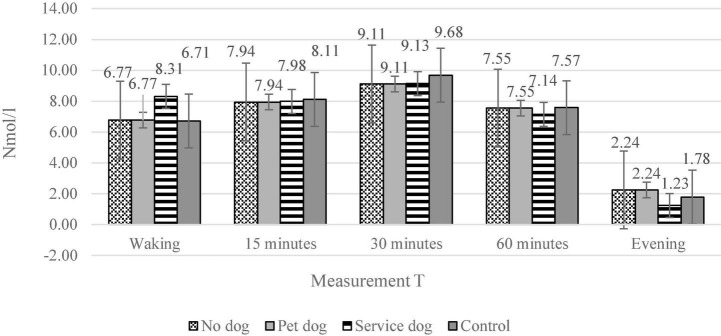
The salivary cortisol levels for the four different subject groups during five different measurement points. The four groups are: individuals with PTSD and a service dog, individuals with PTSD and a companion dog, individuals with PTSD without a dog, Individuals without PTSD. The five measurement moments are: Just after waking up (*n* = 19, 10, 11, 21), 15 min after waking up (*n* = 20, 10, 12, 22), 30 min after waking up (*n* = 20, 10, 12, 22), 60 min after waking up (*n* = 17, 10, 12, 22), just before going to bed in the evening (*n* = 19,10,12,22).

The salivary cortisol levels at all five timepoints were compared between participant groups using a series of Mann-Whitney tests between group pairs. No significant differences were found (Figure 1).

### Activity Differences Between Groups

**FIGURE 2:**
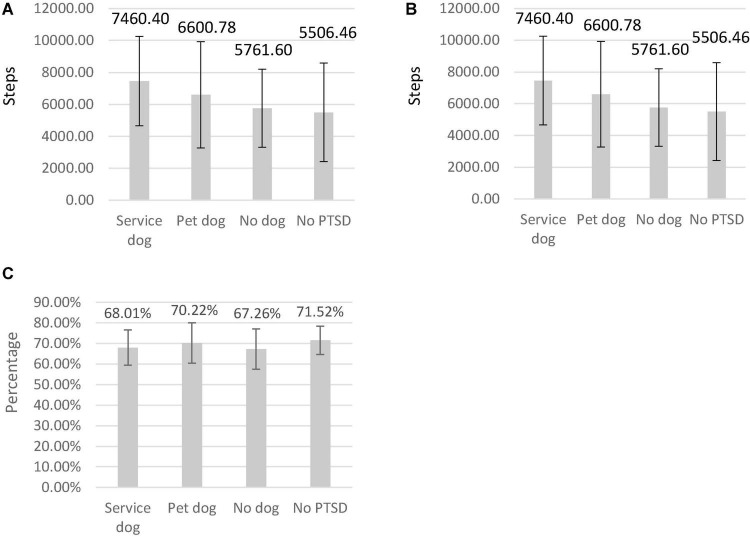
The number of steps taken during 24 h **(A)**, mean step time **(B)**, and percentage stillness **(C)** per participant group. The four groups are: individuals with PTSD and a service dog (*n* = 15), individuals with PTSD and a companion dog (*n* = 9), individuals with PTSD without a dog (*n* = 10), Individuals without PTSD (*n* = 13).

The number of steps taken, mean time walking and percentage of stillness were compared between participant groups using a series of Mann-Whitney tests between group pairs. These tests showed that the total number of steps taken tended to be higher for participants with PTSD and a service dog (Figure 2) than for individuals without PTSD or service dog (*p* = 0.05). No other differences between groups were found.

### Questionnaire Differences Between Groups

**FIGURE 3:**
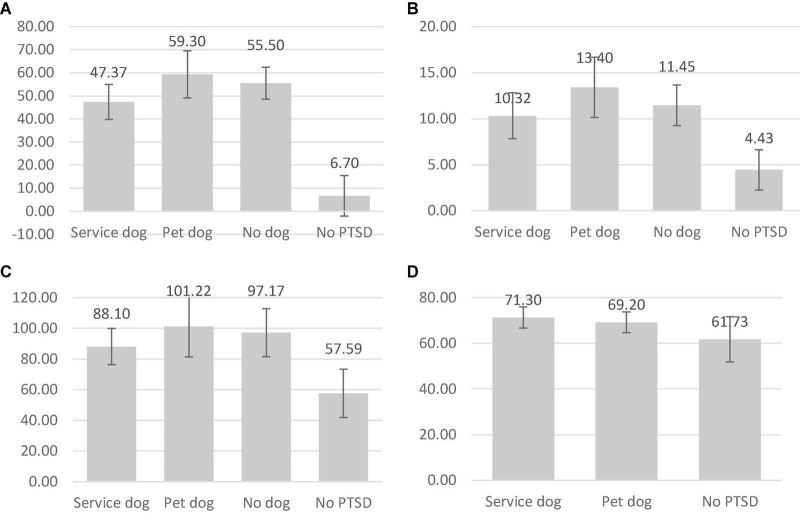
The average scores for the PCL5 **(A)**, PSQI **(B)**, SF36 **(C)**, and MDORS **(D)** questionnaires per participant group. The four groups are: individuals with PTSD and a service dog (*n* = 19, 13, 20, 20), individuals with PTSD and a companion dog (*n* = 10, 9, 9, 10), individuals with PTSD without a dog (*n* = 11, 12, 12), Individuals without PTSD (*n* = 23,22,22,11).

The questionnaire scores of the PCL5, PSQI, and SF36 were compared between participant groups using a series of Mann-Whitney tests between group pairs. Regarding the PCL5 questionnaire, it was found that participants without PTSD or service dog had significantly lower PCL 5 scores (Figure 3A) than participants with PTSD and a service dog (*p* < 0.01, *r* = 0.85), participants with PTSD and a companion dog (*p* < 0.01, *r* = 0.76), and participants with PTSD without a dog (*p* < 0.01, *r* = 0.84). It was additionally found that individuals with PTSD who were supported by a service dog had significantly lower PCL5 scores than those with a companion dog (*p* < 0.01, *r* = −0.51) or with PTSD without a dog (*p* = 0.01).

Regarding the PSQI it was found that participants without PTSD had significantly lower PSQI scores (Figure 3B) than participants with PTSD and a service dog (*p* < 0.01, *r* = 0.78), with PTSD and a companion dog (*p* < 0.01, *r* = 0.76), and PTSD without a dog (*p* < 0.01, *r* = 0.81).

Regarding the SF36 it was found that participants without PTSD had significantly lower SF36 scores (Figure 3C) than participants with PTSD and a service dog (*p* < 0.01, *r* = 0.70), participants with PTSD and a companion dog (*p* < 0.01, *r* = 0.66), and without a dog (*p* < 0.01, *r* = 0.77). It was additionally found that individuals with PTSD who were supported by a service dog had significantly lower SF36 scores than those with a companion dog (*p* = 0.04).

Regarding the MDORS it was finally found that participants without PTSD had significantly lower MDORS scores (Figure 3D) than participants with PTSD and a service dog (*p* < 0.01, *r* = 0.59), and participants with PTSD and a companion dog (*p* = 0.03).

### Relations Between Questionnaire Scores and Other Variables

To see if there was a relation between the PCL5, PSQI or SF36 or MDORS questionnaire scores of participants and either their activity or salivary cortisol measurements, a series of spearman correlations were calculated. These showed that the correlation between cortisol level taken right before an individual went to bed and their PSQI score showed a trend (*p* = 0.07, rho = −0.24). All other correlations were non-significant.

## Discussion

### Subjective Measurements of Service Dog Influence

In our study we found that individuals with PTSD and a service dog had the lowest level of PTSD related symptoms among observed individuals with PTSD. This is in line with earlier studies by Stern and Chur-Hansen (8), Kloep et al. (9), Vincent et al. (10), and Yarborough et al. (11), who all found that individuals with PTSD and a service dog judged their own wellbeing to be better than that of those with PTSD without a service dog. Our study thus demonstrates that individuals with PTSD can experience a service dog as a positive influence on their wellbeing. Because individuals with a pet dog showed more PTSD related symptoms than individuals with a trained service dog, our study furthermore hints that this effect might not an inherent effect of dog presence but a result of service dog training and/or guidance by the organization that provided the service dog. Further study on this topic is still very much needed though to evaluate if this conclusion is true for all service dogs or only those trained by a select number of organizations who follow similar protocols. Additionally it is also possible that those with a service dog suffer from positive bias toward the animal and therefore answered more favorably to questionnaires than those without a service dog. We therefore performed additional measurements to see if the above effects extend beyond the experience of wellbeing and PTSD symptom severity.

### Biological Parameters

The additional measurements performed in this study were measurements of morning salivary cortisol levels and activity levels between groups. Neither of these measurements showed differences between participant groups though, which is interesting when compared to earlier research. Two earlier studies by Rodriguez et al. (14) and Lessard et al. (21) have also studied similar measurements in similar participant groups. Rodriguez et al. (14) studied the influence of service dogs on morning salivary levels while Lessard et al. (21) studied the effect of service dogs on activity levels in those with PTSD. Both studies found an effect of service dogs on these respective measurements which is in contrast to our results. This could be due to the manner in which these parameters were evaluated though. Lessard et al. (21) for example studied activity within individuals while we studied activity between individuals. It is therefore possible that the effect of service dogs on activity levels is small or differs between individuals which makes it easier to measure within than between individuals.

Another possibility for the differences in found results could have been a difference in study populations. In some populations PTSD is known to lower morning cortisol levels (17–20). This was true for the population observed in the study by Rodriguez et al. (14), which made it possible for them to observe an elevating effect of service dog presence on salivary cortisol levels. No lowering of salivary cortisol levels in those with PTSD was observed in our study though, which made it impossible to measure an effect of service dogs on this parameter.

An explanation for why a non-lowered group was observed might be due to a limitation of our study. Out of all approached individuals with PTSD without a service dog, about half of them agreed to participate. The other half stated that they did not feel well enough to participate, and that they wanted to focus on their own recovery instead. Because of these statements it is possible that only individuals with PTSD in relative good welfare participated.

### Correlation Between Subjective and Physiological Parameters

In addition to the above salivary cortisol and activity level as measured in this study also failed to show a correlation with subjective measurements of welfare. Of course it is possible that this was due to the absence of difference within the physiological parameters, though it is also possible that the effects of PTSD on wellbeing and bodily function are truly separate (14). The PTSD service dog itself might for example influence different consequences of PTSD *via* different routes. The activity level of an individual with PTSD for example, might not solely be influenced by the severity of PTSD symptoms and the service dog’s reduction thereof. Measurements of activity level might instead be independently influenced by a dog’s intrinsic need for exercise. This possibility is supported by data found in this study which showed that both those with a service dog and a companion dog walked more than those who did not have a dog. Though this difference was not significant, the absence of a correlation with measurements of wellbeing in this study suggests that dog presence does increase activity in its own right independent of changes in wellbeing.

To accurately measure a correlation between the subjective experience of wellbeing and physiological changes however, repeated measurements over longer periods of time are needed. This was not done within this study, which might also explain a lack of found differences in this variable. It is therefore advisable that future research focusses on repeated measurements over longer periods of time to more accurately establish which effects can be expected of service dogs and whether or not these effects are limited to the experience of wellbeing or can also be measured in physiological parameters.

## Conclusion

In conclusion, our results showed that the presence of a service dog improved the reported quality of life, and lowered the level of reported PTSD symptoms in those with PTSD. No effects of service dog presence were found on activity level and salivary cortisol levels though. These variables were furthermore not linked to measurements of wellbeing. The possibility of bias and placebo that could explain significant differences between the experienced wellbeing of those with and without a service dog effect is therefore difficult to counter *via* our results. Most likely this is due to the used measurement methods which might be more suited for the detection of changes within an individual than they are between individuals. Future research might therefore consider alternative methodology in the study of PTSD service dog effect. Additionally future studies should question though if bias and placebo are truly present in the registration of wellbeing and how big its effect is. Several studies have repeatedly shown a positive influence of service dog presence on those with PTSD. This effect was additionally greater than that of pet dog presence as shown by our results. It can therefore be questioned how big the influence of bias and/or placebo would truly be on our results since similar results have been found across populations. Though the possibility of this influence seems to be small, it is advisable that in future a meta-analysis or similar study is performed on these parameters, to establish a definitive answer to this question.

## Data Availability Statement

The raw data supporting the conclusions of this article will be made available by the authors, without undue reservation.

## Ethics Statement

The studies involving human participants were reviewed and approved by the Medisch Ethische Toetsingscommissie (METC) Utrecht. The patients/participants provided their written informed consent to participate in this study.

## Author Contributions

EH: conceptualization, primary writing, data gathering, and statistics. TR, EV, and NE: conceptualization, review, and funds acquisition. All authors contributed to the article and approved the submitted version.

## Conflict of Interest

The authors declare that the research was conducted in the absence of any commercial or financial relationships that could be construed as a potential conflict of interest.

## Publisher’s Note

All claims expressed in this article are solely those of the authors and do not necessarily represent those of their affiliated organizations, or those of the publisher, the editors and the reviewers. Any product that may be evaluated in this article, or claim that may be made by its manufacturer, is not guaranteed or endorsed by the publisher.
